# ESS: A Tool for Genome-Scale Quantification of Essentiality Score for Reaction/Genes in Constraint-Based Modeling

**DOI:** 10.3389/fphys.2018.01355

**Published:** 2018-09-28

**Authors:** Cheng Zhang, Gholamreza Bidkhori, Rui Benfeitas, Sunjae Lee, Muhammad Arif, Mathias Uhlén, Adil Mardinoglu

**Affiliations:** ^1^Science for Life Laboratory, KTH - Royal Institute of Technology, Stockholm, Sweden; ^2^Department of Biology and Biological Engineering, Chalmers University of Technology, Gothenburg, Sweden; ^3^Centre for Host–Microbiome Interactions, Dental Institute, King's College London, London, United Kingdom

**Keywords:** constraint-based modeling, gene essentiality, genome-scale metabolic models, reaction essentiality, systems biology

## Abstract

Genome-scale metabolic models (GEMs) are comprehensive descriptions of cell metabolism and have been extensively used to understand biological responses in health and disease. One such application is in determining metabolic adaptation to the absence of a gene or reaction, i.e., essentiality analysis. However, current methods do not permit efficiently and accurately quantifying reaction/gene essentiality. Here, we present Essentiality Score Simulator (ESS), a tool for quantification of gene/reaction essentialities in GEMs. ESS quantifies and scores essentiality of each reaction/gene and their combinations based on the stoichiometric balance using synthetic lethal analysis. This method provides an option to weight metabolic models which currently rely mostly on topologic parameters, and is potentially useful to investigate the metabolic pathway differences between different organisms, cells, tissues, and/or diseases. We benchmarked the proposed method against multiple network topology parameters, and observed that our method displayed higher accuracy based on experimental evidence. In addition, we demonstrated its application in the wild-type and *ldh* knock-out E. coli core model, as well as two human cell lines, and revealed the changes of essentiality in metabolic pathways based on the reactions essentiality score. ESS is available without any limitation at https://sourceforge.net/projects/essentiality-score-simulator.

## Introduction

Genome-scale metabolic models (GEMs) are congregations of biochemical reactions that occur in an organism or cell/tissue (Mardinoglu and Nielsen, 2015). GEMs have become one of the denominators in systems biology and have been extensively and successfully used in metabolic engineering, synthetic biology, antibiotic design, understanding diseases, biomarker discovery, and drug target identification (Zhang and Hua, 2015; Benfeitas et al., 2017; Bosley et al., 2017; Uhlén et al., 2017; Mardinoglu et al., 2018; Turanli et al., 2018). GEMs for hundreds of gut microbiota, healthy tissues and tumor samples have been generated in AGORA (Magnúsdóttir et al., 2017), Human Metabolic Atlas (Pornputtapong et al., 2015) and Human Pathology Atlas (HPA) (Uhlén et al., 2015, 2017) efforts, respectively. These resources may provide information for investigating the metabolic capability with species or individual-tumor resolution, but also raise the challenge of comparing and stratifying GEMs in large-scale studies.

There are number of ways to compare GEMs, such as network topology parameters (Doncheva et al., 2012; Vinayagam et al., 2016). For instance, betweenness and degree centrality are two topology parameters frequently used for comparing node importance in a network (Koschützki and Schreiber, 2008). However, topological analyses of GEMs neglect stoichiometric balance and likely miss the required input or output of a metabolic system (Zhang and Hua, 2015). A possible solution would be weighting the stoichiometric properties for each reaction/gene using Essentiality Analysis (EA) (Palumbo et al., 2005, 2007). However, EA only provides binary information about the essentiality of the reaction, and this is insufficient for complex GEMs since there would be too little essential reactions. In this case, a higher degree of EA such as Synthetic Lethality Analysis (SLA) is necessary to provide additional information about non-essential reactions/genes. Generally, it is very difficult to interpret the results of SLA, since thousands to millions of synthetic lethal (SL) combinations may be involved. An option for interpretation of SLA result is using Degree of Essentiality (DoE) (Suthers et al., 2009), but DoE scores ignore the difference between reactions/genes involves in single and multiple SL combinations.

In this study, we present Essentiality Score Simulator (ESS), a tool that uses SLA's results as input to calculate reaction-/gene-specific Essentiality Score (EScore). Unlike topology based methods, ESS relies on the highly valuable biochemical stoichiometric information embedded in GEMs to evaluate the structural properties of reactions/genes. In addition, compared to the DoE, ESS makes the complete use of SLA results and provides higher resolution scores for each reaction/genes.

## Materials and methods

### ESS

The main purpose of ESS is to qualitatively evaluate the essentiality of reactions/genes based on stoichiometric balance. The input for ESS includes a constrain based model either in COBRA (Schellenberger et al., 2011) or RAVEN format (Agren et al., 2013), the objective function and the maximal level of SLA (denoted as level n hereafter) sought by the user. An *n* = 1 implies targeting of a single reaction/gene, whereas *n* > 1 test for combinations of reactions/genes after excluding single lethal reactions/genes. With these inputs, ESS identifies all essential and synthetic lethal reactions/genes internally using an adapted FastGeneSL (Pratapa et al., 2015; Zhang et al., 2015), and then uses a unique algorithm to calculate the ES for each reaction/gene based on the SLA results.

**Figure 1:**
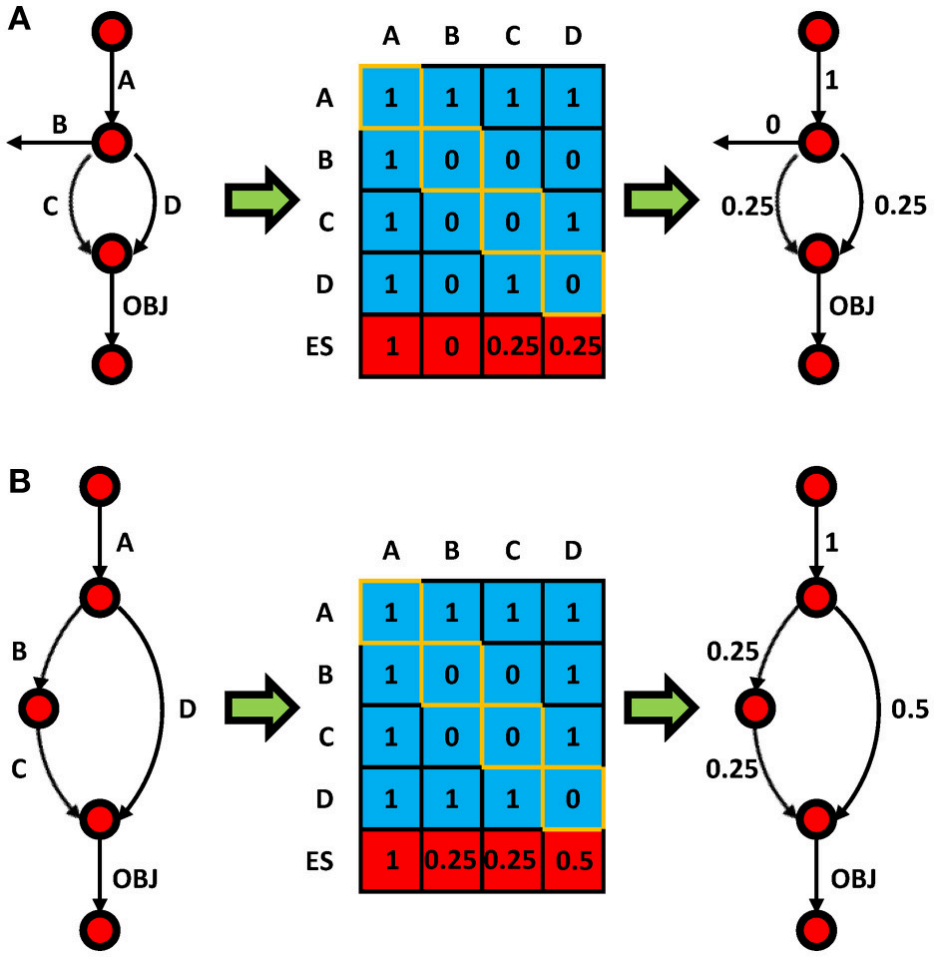
Toy model examples **(A,B)** showing the principal of ESS. Circles and arrows represent metabolites and reactions, respectively. The value in the *i*th row and *j*th column of the blue matrix is 1 if knockout of reaction *i* and *j* lead to zero flux OBJ reaction; otherwise it is 0. Diagonal elements (orange) represent single gene essentialities; all others represent double knockouts. The ES for each reaction (red row) is presented in the matrix and mapped to the pathway (right).

Figure 1 provides examples of level 2 ESS calculations for two sample cases to elucidate the concept. The first pathway (Figure 1A), with 5 reactions and an objective reaction, shows that every non-objective reaction could be essential or synthetic lethal in 4 cases for level 2 SLA (i.e., single C knockout, or double knockouts CA, CB, or CD). For instance, reaction C is not essential *per se*, but double knockout of CD would be essential. As a result of the weighted scores, the ESS of C is 0.25. In this case, only the true SL combinations would be considered in order to eliminate redundancy, and that is why CA was neglected since single deletion of A would be lethal. It should be noted that the EScore for an essential reaction (e.g., reaction A) is always 1 regardless of the level of ESS. Figure 1B shows that reaction D has an EScore that is higher than reaction B and C. This is because reaction D is overrepresented and involved in two SL pairs, while reactions B and C are only involved in one. In other words, ESS could identify the hot-spots in the model. This also explains why reaction D has a higher EScore in Figure 1B than in Figure 1A. As topological view, it can also be explained by the shortest path to the objective function.

It is noteworthy that, this principle could be applied to higher level SLA. The increase of SLA level could give a higher resolution of the EScore. Furthermore, the principle could be implemented for calculation of EScore for genes. To calculate EScore for a specific reaction/gene in any level, all possible synthetic lethal combinations involving a reaction/gene should be introduced for normalization purpose. For example, in the case of the 4 reaction/gene model of Figure 1A, the number of possible combination for reaction/gene A in level 2 ESS is 4 [A(A), AB, AC, and AD]. For level 3 ESS it would then be 16 [A(AA), A(A)B, A(A)C, A(A)D, … AD(A), ADB, ADC, and AD(D)]. Note that, there are repetition in the population [e.g., A(A)D and AD(A)], and this give weight to different combination. Since this number is the same for every reaction/gene in the same model for a given n, we could calculate EScore of a reaction as follows:

$$\begin{array}{l} ES_{i}=\;y_{i1}+\overset{n}{\underset{j=2}{\sum }}\left(\frac{y_{ij}\left(j-1\right)!}{X^{j-1}}\right) \end{array}$$

where *ES*_*i*_ represents the EScore of reaction/gene *i*, y_ij_ represents the number of combination that reaction/gene *i* is SL (or essential) at level *j*. *X* represents number of reactions/genes in the model. In this equation, *ES*_*i*_ for an essential reaction/gene is always 1 since for any *j* > 1, *y*_*ij*_ would be 0; otherwise, *ES*_*i*_ is always no more than 1. In addition, a comparison of EScore among different models is possible since EScore is normalized by the model's *X*. We used level 3 ESS throughout this study considering the balance between accuracy and computational cost.

### Generating the networks and calculation of centrality parameters

Regarding the Bidkhori et al. method (Bidkhori et al., 2018), we generated directed Metabolite-Metabolite network (MMN), directed Reaction-Reaction network (RRN), and directed Gene-Gene network from GEMs (GGN). The currency metabolites (Khosraviani et al., 2016) such as cofactors, coenzymes and H_2_O were removed before generating RRN and GGN. The importance of the nodes (metabolites in MMN, reactions in RRN and genes in GGN) was scored by computing centrality topological parameters i.e., betweenness, eccentricity, closeness, and degree (Bidkhori et al., 2018). The nodes were ranked in the basis of each node's topological measures. The ranking and scoring were calculated for each node in the largest connected component in each generated network.

### Experimentally measured cell line essentiality scores

Genome-wide CRISPR gene essentiality scores (CERES) were retrieved from Avana public datasets 18Q2 (Meyers et al., 2017). In brief, essentiality of each gene in cancer cell lines were tested using lentiviral vectors expressing the Cas9 nuclease and calculated by a computational method developed in that study. The CERES scores are used to benchmark the computationally estimated EScore in this study.

### Model and simulation

The *E. coli* core model was reconstructed based on a previous study (Orth et al., 2010) and retrieved from BiGG database (King et al., 2016). The original constraints and objective function are used for simulations of wild-type E. coli. The model for ldh knockout E. coli mutant is obtained by removing the reaction “LDH_D” in the wild-type model. The EScore of “LDH_D” for mutant model is set the same as in wild-type model for fair comparison with the wild-type model. The reaction “ATPM” is excluded from the ESS calculation since it is a pseudo reaction that presents a mandatory function of the model.

Cell line specific GEMs, iIPC298 and iNCIH1299, were reconstructed based on the RNAseq gene expression data downloaded from CCLE public dataset 18Q2 as RPKM (Barretina et al., 2012) and using a previously developed task-driven model reconstruction (tINIT) algorithm in RAVEN toolbox (Agren et al., 2014). The tINIT algorithm employs defined metabolic tasks for imposing constraints on the functionality of the reconstructed models. In this specific case, we changed the input of the growth task from Ham's media to RPMI1640 which was used in the experiments. The constraints for the cell GEMs calculated based on the RPMI1640 composition are provided as supplementary (Table S1). The adapted metabolic tasks, the RNA-Seq data and a generic GEM for human cancer from our previous study (Uhlén et al., 2017) were used as inputs for the tINIT algorithm, and as a result, cell line specific GEMs have been generated.

All linear programming and mixed integer linear programming problems are solved by the freely available “mosek” solver version 8 obtained from https://www.mosek.com/downloads/. All computational times reported in this paper are for a single core CPU with 6 GB RAM in server.

### Availability

ESS is a method build in Matlab (version R2017b) environment, and the source code and models (including the cell line models) are available at https://sourceforge.net/projects/essentiality-score-simulator/. In addition, SBMLlib (Bornstein et al., 2008) and COBRA/RAVEN toolbox (Schellenberger et al., 2011; Agren et al., 2013) are also required to import SBML format GEMs.

## Results

### Case 1: benchmark ESS using *E. coli* core model

To demonstrate the advantage of ESS compared to topology based method, we firstly employed the core metabolic model of *E. coli* with 95 reactions as a proof of concept case. We calculated and compared the EScores and betweenness centralities (BCs) which is one of the most used topology based method for all reactions in the core model (Table S2). Within the 95 reactions, there are 10 reactions that display zero scores for both EScores and BCs, and 49 reactions that have non-zero scores for both EScores and BCs. Spearman correlation between EScores and BCs for the 49 reactions was found substantially low (*r* = 0.0994; *P* > 0.1), suggesting inconsistency between two metrics although both methods classify these reactions as essential to a certain extent. There are 16 reactions that have non-zero EScores and zero BCs, and 15 of them are responsible for exchanging of very important or essential metabolites, including carbon dioxide, oxygen, glucose, ethanol, formate, lactate, proton, ammonium, phosphate, and succinate. Moreover, among these exchange reactions, those for glucose, proton and ammonium exchange have the maximum EScores (equal to one), which means they are essential for growth of *E. coli* in the given condition. This highlights the limitation of topology based methods which missed uptake of glucose as essential because of neglecting stoichiometric info as previously reported (Zhang and Hua, 2015). In addition, there is also another reaction, RPI (ribose-5-phosphate isomerase), that is also missed by betweenness (BC = 0), and this reaction has been already experimentally validated as essential in *E. coli* in previous study (Neidhardt and Curtiss, 1999) which further proves the advantage of ESS compared to betweenness. On the other hand, there are 20 reactions that showed non-zero scores uniquely in betweenness analysis. These reactions are mainly involved in glycolysis and TCA cycle, including FBP (fructose-bisphosphatase), AKGDH (2-oxogluterate dehydrogenase) and SUCOAS (succinyl-CoA synthetase). The observation that other reactions show both significantly higher BCs and EScores (Student *t-test*; *P* < 0.05) suggests that these 20 reactions are possibly not metabolically important. Moreover, some of them have been reported to be non-essential experimentally, which is the case of FBP in *E. coli* (Fujita et al., 1998).

### Case 2: benchmark ESS using human cell line GEMs

In order to further test the efficiency and accuracy of ESS in actual genome-scale model cases, we reconstructed two novel human cell line specific GEMs for IPC298 and NCIH1299, named iIPC298 and iNCIH1299, respectively. We selected these two cell lines based on availability of transcriptomic data and experimentally derived CRISPR essentiality scores (CERES algorithm, see section Materials and Methods), and both are cultured in the same medium, RPMI1640. The iIPC298 includes 3414 reactions, 2102 metabolites, and 1827 genes, whereas iNCIH1299 includes 3550 reactions, 2117 metabolites and 1906 genes. Then, we applied level 3 ESS to these two human cancer cell line GEMs to calculate EScores for genes. The computational costs for this analysis were much lower compared to greedy search which was used in most EA analysis (Table 1). We also applied several frequently used topology methods to these two GEMs including Betweenness Centrality, Closeness Centrality, Eccentricity Centrality and Degree (Table S3). To check how much ESS can identify experimentally derived gene essentialities, we calculated the Spearman's correlation between all *in silico* predicted essentiality or centrality scores and the CRISPR derived CERES scores for all genes with non-zero EScores (Table 2).

**Table 1:** Computational costs of ESS and greedy search for level 3 synthetic lethality analysis for human cell line GEMs.

**Tested GEMs**	**Benchmark methods**	
	**ESS**	Greedy search^*^
iIPC298	29.3 h	704.7 d
NCIH1299	45.0 h	800.1 d

* *Computational cost for greedy search are estimated based on number of linear programs needed, and each linear programs takes 0.06 s in the estimation*.

**Table 2:** Spearman correlation between experimentally derived CERES scores and the *in silico* methods.

**Methods**	**iIPC298**		**iNCIH1299**	
	**Correlations**	***P*-values**	**Correlations**	***P*-values**
Essentiality scores	−0.194	0.014	−0.205	0.007
Betweenness centrality	−0.074	0.354	−0.138	0.070
Closeness centrality	−0.026	0.746	−0.065	0.396
Eccentricity centrality	−0.046	0.563	−0.011	0.883
Degree	0.070	0.376	−0.009	0.911

CERES scores are normalized essentiality scores of genes where the median values for essential and non-essential genes are −1 and 0, respectively. Therefore, the correlation between EScores and CERESs should be negative if they agree with each other. Indeed, the respective correlations between EScores and CERESs for iPC298 and iNCIH1299 are −0.194 and −0.205, and both of them being statistically significant (*P* < 0.05), whereas, as shown in Table 1, the correlations between network topology based parameters for these genes and CERESs for iPC298 or NCIH1299 could not meet the same statistical significance. These suggest that the EScores calculated by ESS are accurate and correlate better with experimental data compared to topology based method.

### Case 3: comparison between wild-type and LDH knockout *E. coli* core model

As a proof of concept, we applied the method to the core metabolic model of *E. coli* of wide-type and *ldh* knockout mutant and calculated the EScores for all reactions in both models to demonstrate the usage of ESS in model comparison. The full results of level 3 ESS for wild-type and mutant E. coli core model are provided as Table S4. First of all, we found 48 and 4 reactions which respectively display larger and lower EScores in the knockout, and the average EScore is increased after *ldh* knockout. This suggests that the mutant metabolic model is less robust in terms of growth, which is expected since deletion of metabolic genes probably decreases the metabolic potential of the bacteria. Moreover, the metabolic pathways with the highest increases in EScore increases are acetaldehyde dehydrogenase (ACALD), oxidative phosphorylation (NADH16 and CYTBD), uptake of oxygen (EX_o2_e and O2t) and formate transportation (FORt2 and EX_for_e). ACALD and formate transportation are key alternatives for LDH_D in the model which explains these EScore increases. In addition, LDH_D is an important reaction for anaerobic growth, and therefore its deletion will surely increase the importance of aerobic metabolic function. This explains why oxidative phosphorylation and oxygen uptake have the highest increase in EScore since they are key pathways in aerobic growth for *E. coli*. On the other hand, the reactions with top EScore decreases are D_LACt2 and EX_lac__D_e, both of which are responsible for transportation of lactate. These two reactions are downstream of LDH_D in the model, and their EScores decreased since they become dead-end reactions in the mutant model.

## Discussion

In this study, we proposed a novel method name ESS, which shows as an accurate and efficient method for quantifying reaction and gene EScores. This method was implemented in Matlab and that requires standard constraint-based modeling toolboxes (COBRA or RAVEN). While gene and reaction essentiality have extensively been examined using GEMs, we here introduce a comprehensive scoring system for determining essentiality for single and combined reactions or genes. ESS employs logical transformation of model to refine the gene-protein-reaction relationships, avoiding the potential problem caused by redundant enzymes during the essentiality analysis. We benchmarked ESS against topology based methods and using experimentally derived essentiality scores for reference pan-essential and non-essential genes, and observed that ESS displays higher accuracy than topology-based methods. It should be noted that essentiality in GEMs is dependent on the inputs and objective function. Therefore, EScores could be different for the same GEMs given different inputs and/or objective function. As we showed in the last case study, the proposed method could be easily used to compare GEMs generated from the same reference model. Therefore, it could be a promising tool for future studies that investigate the relationships among gut microbiota, different cells and tissues using resources such as AGORA and HPA where models were generated from a common reference. In this context, we are confident that ESS will have a great application in biotechnology and systems medicine.

## Author contributions

AM and CZ conducted the study. CZ developed the method. SL, RB, GB, MA and MU helped in development of the method. CZ performed most of the analysis and GB helped in the analysis. CZ wrote the paper and all authors were involved in editing the paper.

### Conflict of interest statement

The authors declare that the research was conducted in the absence of any commercial or financial relationships that could be construed as a potential conflict of interest.

## Supplementary material

Table S1 The contraints for exchange reactions to simulate the input from RPMI medium.

Table S2 EScores and BCs for *E. coli* core model.

Table S3 CERES, EScores and all other topological parameters calculated for cell line specific GEMs.

Table S4 The full results of level 3 ESS for wild-type and mutant *E. coli* core model.
